# Characterization of the Anisotropic Electrical Properties of Additively Manufactured Structures Made from Electrically Conductive Composites by Material Extrusion

**DOI:** 10.3390/polym16202891

**Published:** 2024-10-14

**Authors:** Maximilian Nowka, Katja Ruge, Lukas Schulze, Karl Hilbig, Thomas Vietor

**Affiliations:** Institute for Engineering Design, Technische Universität Braunschweig, Hermann-Blenk-Str. 42, 38108 Brunswick, Germanylukas.schulze@tu-braunschweig.de (L.S.); k.hilbig@tu-braunschweig.de (K.H.); t.vietor@tu-braunschweig.de (T.V.)

**Keywords:** anisotropic electrical resistivity, conductive polymer composite (CPC), electrically conductive, additive manufacturing (AM), material extrusion (MEX), 3D printing, fused deposition modelling (FDM), commercial filament, scanning electron microscopy (SEM), composite material

## Abstract

Additive manufacturing (AM) of components using material extrusion (MEX) offers the potential for the integration of functions through the use of multi-material design, such as sensors, actuators, energy storage, and electrical connections. However, there is a significant gap in the availability of electrical composite properties, which is essential for informed design of electrical functional structures in the product development process. This study addresses this gap by systematically evaluating the resistivity (DC, direct current) of 14 commercially available filaments as unprocessed filament feedstock, extruded fibers, and fabricated MEX-structures. The analysis of the MEX-structures considers the influence of anisotropic electrical properties induced by the selective material deposition inherent to MEX. The results demonstrate that composites containing fillers with a high aspect ratio, such as carbon nanotubes (CNT) and graphene, significantly enhance conductivity and improve the reproducibility of MEX structures. Notably, the extrusion of filaments into MEX structures generally leads to an increase in resistivity; however, composites with CNT or graphene exhibit less reduction in conductivity and lower variability compared to those containing only carbon black (CB) or graphite. These findings underscore the importance of filler selection and composition in optimizing the electrical performance of MEX structures.

## 1. Introduction

Additive manufacturing (AM) enables the production of components with complex geometries and material combinations that are challenging or impossible to create using conventional manufacturing methods. One of the most widely used AM technologies is material extrusion (MEX) [1], also known as fused deposition modeling (FDM), which typically processes thermoplastic materials by extruding them through a heated nozzle. MEX allows for the design of multi-material parts [2,3,4,5], enabling the precise tailoring of material properties to meet specific functional requirements. Integrating electrical conductivity into AM through the use of components made of electrically conductive polymer composites (CPCs) has become increasingly important for applications such as sensors [6,7,8,9,10,11,12,13,14,15,16,17,18,19,20], actuators [21,22,23,24], energy storage devices [25,26,27,28], and electrical interconnects between other electrical functional structures [25,29,30,31,32,33,34].

CPCs composed of a thermoplastic matrix embedded with conductive fillers like carbon nanotubes (CNTs), graphene nanoplatelets (GNPs), carbon black (CB), or metal particles, impart electrical conductivity to otherwise insulating polymers. The fillers can be blended into the matrix polymer using a polymer solvent by solution mixing [35,36,37,38,39,40] or by melt-blending with extruders, internal mixers, or molding machines [15,33,36,41,42,43,44,45,46,47]. The electrical properties of additively manufactured parts made of CPCs are influenced not only by the fillers themselves or the mixing process but also by the AM process, particularly through parameters such as extrusion temperature, build platform temperature, and deposition speed [14,17,21,37,48,49,50,51]. Among these factors, path planning, the strategy for the selective material deposition, plays a significant role in the resulting anisotropic electrical properties of the final part [21,22,23,48].

Anisotropy in electrical properties refers to the variation in resistivity depending on the direction of the individual depositioned material, so-called strands, relative to the current flow. In MEX, anisotropy is caused by the selective deposition process, where orientation of strands relative to a reference system is controlled by path planning. This process can significantly affect the electrical properties of printed components, with resistivity varying depending on the direction of current flow—parallel, perpendicular to the strands, or through the layers [21,22,32,48,52,53]. Anisotropy is of particular importance when designing mechatronic components, such as sensors and actuators, with particular specifications in mind. Therefore, path planning must account for the intended use of the component to optimize its electrical properties.

Several studies have demonstrated how path planning in relation to the mechanical or electrical load direction influences the performance of sensors and actors. For example, Kim et al. produced a three-axis piezoresistive force sensor that exhibited varying sensitivities due to the orientation of material strands within the xy-plane and along the z-direction [54]. Similarly, Goutier et al. and Dul et al. identified a correlation between infill angle and the sensitivity of piezoresistive sensors, attributing this to the weakness of the network at the contact points between welded strands [6,55]. Watschke et al. and Hilbig et al. observed anisotropic heat distribution in resistive heating structures (Joule effect) with different fill patterns, demonstrating the impact of orientations other than 0° and 90° on performance [21,22]. Dijkshoorn et al. specifically used path planning to create a structure that concentrated current in the center [56]. These examples illustrate the significant influence of path planning on anisotropic properties, especially in systems subject to mechanical and electrical stress.

Despite the availability of conductive composites for MEX, there is limited detailed information about their electrical conductivity, largely due to gaps in manufacturer data. Additionally, the characterization methodologies in research and manufacturer specifications are inconsistent. Most studies have focused on other aspects than resistivity (DC, direct current), leading to different specimen geometries, inconsistent measurement methods, or poor-quality manufacturing. For example, Lazarus et al. and Watschke et al. measured rectangular bars without compensating for contact resistance, which affected their results [21,57]. Stankevich et al. analyzed the resistivity of filament, extruded fibers, and MEX-printed specimens, providing more comprehensive data [53]. Flowers et al. measured the resistivity of three composites using zigzag-shaped specimens with square contact surfaces but provided little information on the resistance measurement method [25]. Overall, there remains a gap in the literature regarding detailed measurements of anisotropic electrical conductivity in MEX components.

This study aims to address these gaps by systematically characterizing the anisotropic electrical conductivity of MEX structures made from 14 commercially available conductive filaments in a comparable manner. The resistivity of CPCs will be examined at three stages: as unprocessed filament feedstock, as extruded fibers, and as fully fabricated MEX structures. The objective of this study is to analyze the effect of conductive fillers on the electrical conductivity at each stage and the resulting anisotropy from the MEX process. This will provide a database for the selection of CPCs based on anisotropic resistivity, as well as a basic model for explaining the underlying cause.

### 1.1. Material Extrusion

Material extrusion ranks among the most prevalent methods for the AM-fabrication of multi-material components from thermoplastics through additive manufacturing [1]. The material extrusion process (MEX) of thermoplastic polymer (P) feedstock is specified as MEX-TRB/P, in accordance with the ISO/ASTM 52900 standard. In this process, the feedstock is applied via material extrusion through a nozzle. The layer is created through the principle of a thermal reaction (TRB). No further, finer classification is made with regard to the type of feedstock (e.g., powder, granulate, wire) used or the subsequent feeding mechanism [58]. In this investigation, a polymer wire, the filament, is used as feedstock. The filament is plasticized in the heated nozzle and pressed out of the nozzle by continuous feeding. Immediately after exiting the nozzle, the melt welds together with the neighboring or underlying strands and then solidifies [2,59,60].

### 1.2. Resistivity in Context of Material Extrusion

The resistance R of a MEX structure is dependent on its geometry and therefore unsuitable for comparison of material properties. In contrast, the resistivity ρ is independent of geometry and is calculated from the conductor length L and the electrically conductive cross-sectional area A. In the context of material extrusion, the production parameters P impact the structural resistance R_P_ and thus the resistivity ρ*_P_*:

(1) $$\varrho_{P}=R_{P}\cdot \frac{A}{L}=\frac{1}{\sigma_{P}}$$

**Figure 1:**
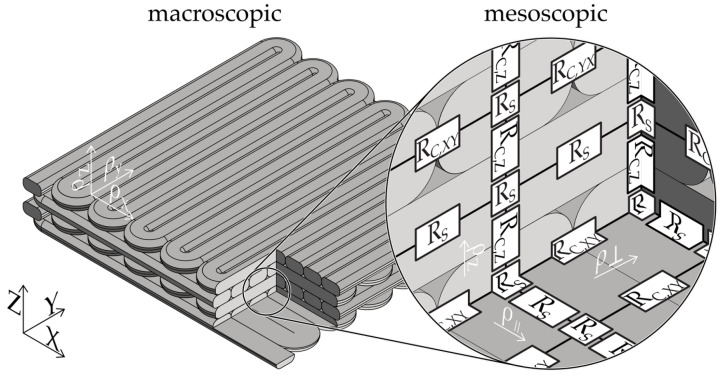
Simplified model of the resistances within an additively manufactured electrically conductive structure. Macroscopic resistivity for x, y, and z direction: ϱ_x_, ϱ_y_, ϱ_z_ = mesoscopic resistivity depending on the infill pattern: ϱ_∥_ = for parallel, ϱ_⊥_ = orthogonal. R_C, XY_ = intralayer contact resistance between strands, R_C,Z_ = interlayer contact resistance between strand in different layers.

The inverse of the resistivity is the conductivity σ*_P_*. The anisotropic resistivity of additively manufactured structures is a consequence of their strand and layered manufacturing. The observed effect can be attributed to the contact resistance resulting from the presence of a less-conductive skin layer due to an inhomogeneous filler distribution between the welded strands [32,52,53,61] and lower cross-sectional interface area [42]. Figure 1 illustrates an exemplary structure observed at both macro- and mesoscopic levels, featuring multiple layers offset by 90°.

The electrical conductivity of additively manufactured conductive structures can be described at three distinct levels: the macroscopic component level, the mesoscopic path planning level, and the microscopic material level.

At the macroscopic level, the resistance is defined by the geometry of the functional structure and the anisotropic resistivity at the component level (ρ_x_, ρ_y_, ρ_z_). The macroscopic resistivity can be derived from the superposition of mesoscopic resistivities in each layer (ρ_∥_, ρ_⊥_) and between layers (ρ_z_). In this model, the resistivity in the build direction is not affected by the path planning (filling pattern), such that the resistivity ρ_z_ remains identical at both the mesoscopic and macroscopic levels.

The mesoscopic resistivity gradient of a layer is determined by the orientation of the strands in relation to the current flow. The resistivity in the direction of the current flow, ρ∥, can be derived exclusively on the basis of the strand resistivity, R_S_. In contrast, the resistivity orthogonal to the strand direction (ρ_⊥_) is influenced by both the strand resistance (R_S_) and the intralayer contact resistance (R_C,XY_). The resistivity in the z-direction, ϱ_z_, is determined by the strand resistance, R_S_, and the interlayer contact resistance, R_C,Z_. The resistances at the level of the strands (R_S_, R_C_, Z, R_C,XY_) are particularly susceptible to alterations due to the composition of the material and its processing, including MEX processing at the microscopic level.

## 2. Materials and Methods

**Figure 2:**
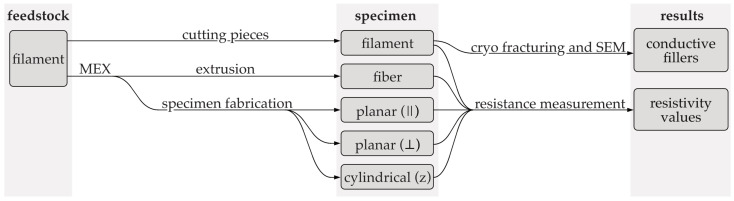
Schematic overview of the specimen preparation and characterization process. ∥ = current flow in xy-plane parallel to strands, ⊥ = current flow in xy-plane perpendicular to strands; z = current flow in z-direction.

The objective of this study was to measure the resistivity of commercially available composites at various stages of the production process, in order to gain insight into the effects of processing on their resulting electrical properties. The methodology employed in this study is illustrated in Figure 2.

The specimens for the DC resistance measurement included the following:

1. Filament with a nominal diameter of 1.75 mm.
2. Fibers with a nominal diameter of 400 µm extruded into the air with a MEX machine.
3. Rectangular planar monolayer MEX specimen (60 × 24 × 0.2 mm) for the xy-plane.
4. Cylindrical hollow single wall (⌀7.64 × 62) MEX specimen for the z-direction.

The interpretation of measurement data was supported by analysis of scanning electron microscope (SEM) images. This enabled a comparison of the conductive fillers present with the manufacturer’s specifications and the identification of any additional fillers.

### 2.1. Conductive Polymer Composites for Material Extrusion

This study examined 14 commercially available conductive polymer composites that can be classified as electrically conductive, with a resistivity order of magnitude less than or equal to 10^3^ Ωcm [62,63,64]. It is not feasible to investigate the use of electrically dissipating composites as an electrical functional structure due to their lack of conductivity. The composites are available with a variety of conductive fillers, including carbon black (CB), graphene (Gr), carbon nanotubes (CNT), carbon fiber (CF), graphite (G), and copper particles (CuP). The matrix polymer of the majority of the composites is a standard thermoplastic polymer, such as polylactic acid (PLA) or polycaprolactone (PCL). A small number are based on thermoplastic polyurethane (TPU), which is an engineering plastic, while only one utilizes a high-performance polymer, namely polyvinylidene fluoride (PVDF). Table 1 provides a list of commercially available electrically conductive filaments that are examined as part of this study.

The MEX specimens are manufactured using the Toolchanger material extrusion system (E3D-Online, Chalgrove, Oxfordshire, UK). The system is equipped with Hemera direct drive filament extruders (E3D-Online, Chalgrove, Oxfordshire, UK) and hardened coated 400 µm Nozzle X nozzles (E3D-Online, Chalgrove, Oxfordshire, UK). Prior to MEX processing, the filaments were subjected to a drying period of at least 48 h (PLA at 60 °C, PCL at 30 °C, TPU and PVDF at 80 °C). Table 2 presents a summary of the process parameters used in the production of MEX specimens.

The process parameters for sample production, as outlined in Table 2, are defined through the following methodology. The mean value of the process parameter window was employed. In the event of contradictory information (datasheet, information on filament spool), the process parameter window on the filament spool was used for the calculation (for further details, please refer to the overview of process parameters in Table A1). In the absence of specified process windows for parameters, the process parameters of similarly composed composites were used.

Additional exceptions were made for the following materials due to material properties: Due to the low shore hardness of the TPU-based composites and the resulting demanding processing, the highest processing temperature and the lowest deposition speed were used. The conductivity of the PCL/Cu composite material, Electrifi, was significantly reduced as a result of temperature-induced oxidation [21,65]. Therefore, the lowest extrusion temperature was selected. The thin-walled z specimens were produced at a speed of 10 mm/s (5 mm/s for Electrifi) to ensure complete solidification of the previous layer. Table A1 provides an overview of the process parameter windows and the selected process parameters.

The use of electrical bonding agents is a common practice in the reduction of contact resistance, with the necessity of considering adhesion in the selection process [21,22,23,24,25,34,79]. Colloidal silver EMS #12640 (Electron Microscopy Sciences, Hatfield, PA, USA) is the preferred bonding agent. In instances where adhesion cannot be achieved, silver epoxy 8331D-14G (MG Chemicals Ltd., Burlington, ON, Canada) is used as an alternative. However, it should be noted that neither of the aforementioned bonding agents adheres to the composite Eel 3D-Printer Filament in the form of filament. Therefore, the measurement of this filament was performed without a bonding agent.

The manufacturing parameters, comprising extrusion speed and temperature of the fiber specimens, were derived from the process parameters of the planar specimens and, as a consequence, are not included in Table 2. The extrusion temperature was identical to that employed in the manufacturing of the MEX samples. To ensure comparability of the shear conditions in the nozzle with those of the MEX specimen manufacturing, the volume flow was set to the same rate.

### 2.2. Scanning Electron Microscopy

**Figure 3:**
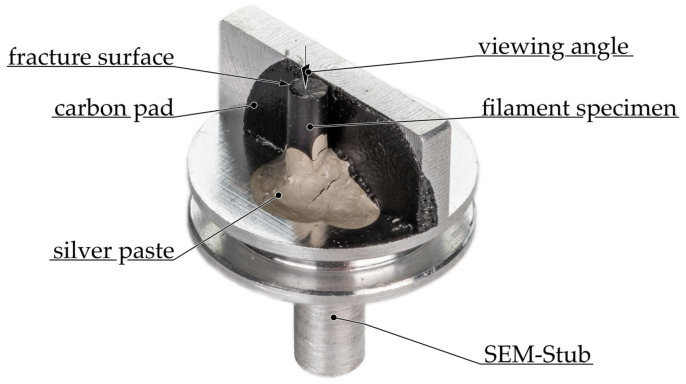
Preparation of cryo-fractured filament specimen for SEM imaging.

The precise composition of the composites is considered a trade secret; therefore, scanning electron microscopy (SEM) images of the filaments were taken to identify the actual fillers they contained. As part of the specimen preparation process, the filament was initially cooled below the glass transition temperature with liquid nitrogen and subsequently fractured in a brittle manner. The samples were mounted with the fracture surface facing upwards on an SEM holder with a carbon pad and colloidal silver (EMS#12640). An example of a prepared specimen is illustrated in Figure 3.

All specimens were coated with a 4 nm thick layer of platinum by sputtering (EM ACE600, Leica Microsystems, Wetzlar, Germany) to enhance charge transport. The images were recorded with a Helios G4 CX (Field Electron and Ion Company, Hillsboro, OR, USA) at 3 to 5 keV with the secondary electron detector.

### 2.3. Resistivity Measurement

The filament was examined as a reference to obtain an indication of the extent to which the resistivity changed as a result of the MEX process. Individual fibers were extruded into the air to serve as a reference value for the influence of the extrusions. In order to account for the anisotropic electrical component properties of MEX structures, it was necessary to separately measure the electrical material properties in the three directions. In order to achieve this, two specimen variations were created, with the aim of determining the resistivity in both the xy-plane and the z-direction. The following section provides a more detailed description of the electrical boundary conditions for the measurement method and the specimens.

#### 2.3.1. Electrical Boundary Conditions for the Determination of Resistivity

The determination of the resistivity was based on the standard ISO 3915:2022-5, which covers the determination of the resistivity of electrically conductive plastic composites with a resistivity of less than 10^6^ Ωcm [80]. The specified electrical boundary conditions apply to all the electrical measurements in this study and are identical regardless of the specimen geometry.

The resistance measurements were conducted with a Keithley 2460 source meter (Keithley Instruments, Solon, OH, USA) in a four-wire configuration. This approach enabled the compensation of two force lead resistances, R_FL1,2_, and two sense lead resistances, R_SL1,2_. The use of electrical conductive contacts, created through the application of an electrical bonding agent, established a precise contact area for the 4-wire measurement; see Table 2 [21,22,23,24,25,34,79]. The 4-wire measurement was conducted with the use of separate cables for the current supply and for the measurement of the voltage drop. This methodology was employed to prevent the distortion of measurements that would otherwise occur in a 2-wire measurement, due to the influence of wire resistances and contact resistances. The contact surface geometry was created by applying the bonding agent to the specimen with a mask made of polyimide tape (Kapton, DuPont, Wilmington, DE, USA). Accordingly, the distance between the contact points was known, with an error of less than 0.2%. The contact mediator was left to dry for a minimum of 24 h prior to the measurements. The measuring current of 100 µA was fed via the two outer contacts, and the resulting voltage drop was measured via the two inner contacts. The small measurement current of 100 µA limited, in combination with the measurement voltages (<50 V), the power dissipation to below 100 mW.

Any specimens manufactured by AM that deviated more than ± 5% from the specified geometry at one or more measurement points were considered unacceptable and replaced with acceptable specimens. All measurements were conducted at a temperature of 23 ± 1 °C.

#### 2.3.2. Specimen and Measurement Setup for Measuring the Resistivity of Filament

**Figure 4:**
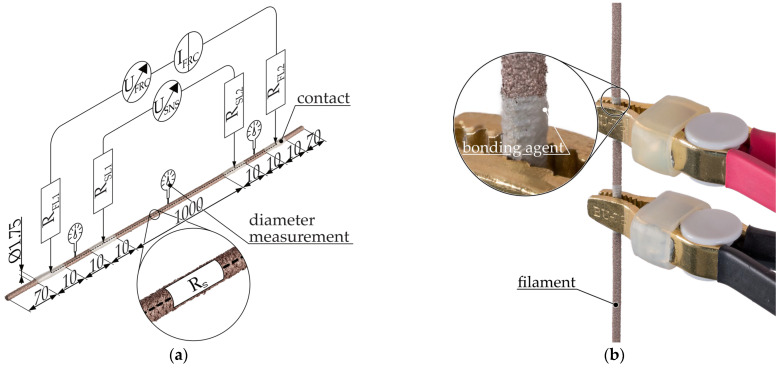
Measurement setup for filament specimens: (**a**) filament specimen with superimposed schematic representation of the resistance measurement setup using 4-wire measurement for determining the resistivity; (**b**) single-sided representation of the filament specimen with connected measuring terminals. R_S_ = resistance of the sample, R_FL_= resistance force lead, R_SL_ = resistance sense lead, I_FRC_ = forced current, U_FRC_ = voltage needed to force current, U_SNS_ = measured voltage drop across specimen.

The specimens for determining the resistivity in the filament ϱ_fila_ were approximately 1200 mm long pieces of filament [24]. Figure 4a illustrates a shortened specimen with a superimposed measuring circuit.

The filament diameter is measured with a micrometer screw (QuantuMike^®^ 293-140-30, Mitutoyo Corporation, Kawasaki, Japan) in the immediate vicinity of the electrical contacts and in the center before application of the bonding agent (see marked spots in Figure 4a). The contacts from the electrical contact mediator are applied as a circumferential cylindrical surface. Two pairs of terminals are connected to the contacts as illustrated in Figure 4b. Five filament specimens are then measured for each composite.

#### 2.3.3. Specimen and Measurement Setup for Measuring Fiber Resistivity

**Figure 5:**
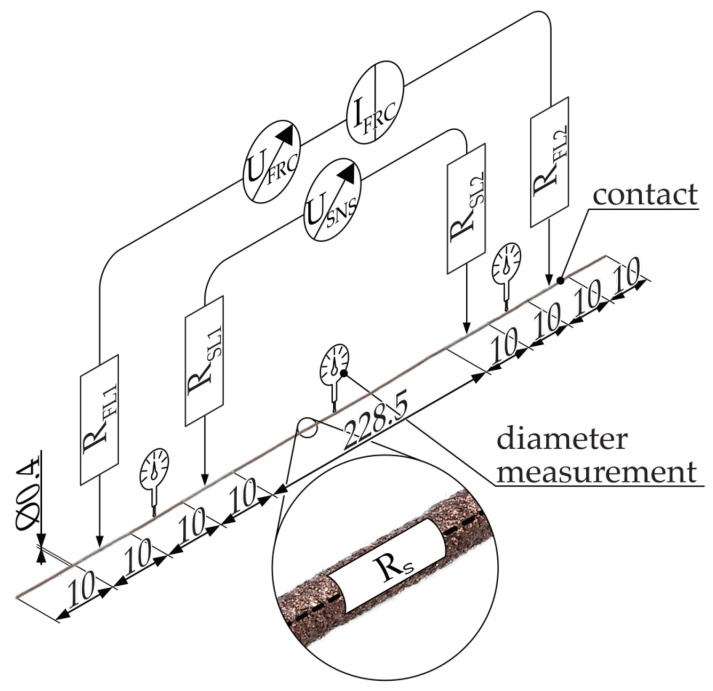
4-wire resistance measurement setup for fiber specimens. R_S_ = resistance of the specimen, R_FL_ = resistance force lead, R_SL_ = resistance sense lead, I_FRC_ = forced current, U_FRC_ = voltage needed to force current, U_SNS_ = measured voltage drop across specimen.

In order to evaluate the impact of the extrusion process on the resistivity, the filament was extruded by the extruder into the air, resulting in the formation of fiber with a nominal diameter of 400 µm. The nozzle was positioned above the building platform to prevent contact between the viscous polymer melt and the platform. Prior to extruding the specimen, the entirety of the polymer melt within the nozzle was purged. This step was undertaken to prevent the specimen from being affected by thermal effects, such as degradation, which can occur if the material remains in the nozzle for an extended period of time. The specimens were comparable to the filament specimens in terms of their composition and dimensions. The distance between the measuring contacts was 228.5 mm, ensuring a consistent length-to-diameter ratio as used for the filament specimen. The width and distance between the contact areas were both 10 mm. Figure 5 illustrates the configuration of a fiber specimen.

The procedure for measuring the diameter of the fiber, applying the contact agent, and measuring the resistance was identical to that used for the filament specimen. Seven fiber specimens were produced and measured for each composite.

#### 2.3.4. Specimen and Measurement Setup for Measuring Planar MEX-Resistivity (xy)

**Figure 6:**
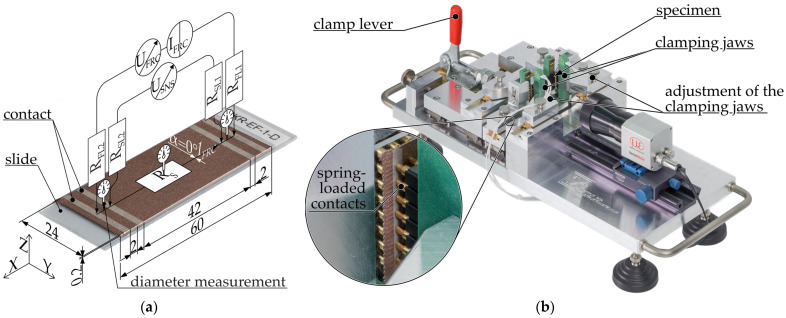
Measurement setup for planar specimens: (**a**) planar MEX-specimen (infill orientation α = 0°) with superimposed schematic representation of the resistance measurement setup using 4-wire measurement for determining the resistivity in the xy-plane; (**b**) planar Multi3d Electrifi specimen clamped in test rig. R_S(P)_ = resistance of the specimen depending on the MEX parameters, R_FL_ = resistance force lead, R_SL_ = resistance sense lead, I_FRC_ = forced current, U_FRC_ = voltage needed to force current, U_SNS_ = measured voltage drop across specimen.

The specimens employed for the measurement of resistivity within layers, designated as ρ_∥_ and ρ_⊥_, were rectangular, 200 µm-thick monolayers without contour paths and were produced on a microscope slide. Once the specimen was produced, it was not removed from the slide. [24] The specimens were manufactured with an infill orientation of α = 0 (ρ_∥_) and 90° (ρ_⊥_) relative to the flow direction of the measuring current I_FRC_. In the case of the 90° filling pattern orientation, the measuring current must have passed the contact resistance between the strands running orthogonally to the current flow. The number of contact points was dependent upon the distance between the two measuring contacts and the track width. In contrast, in the case of a 0° fill pattern orientation, the strands ran continuously from one measuring contact to the next, such that the contact resistance between two strands was not a factor. It should be noted, however, that the track width [53] and the layer height [51,79,81] may have also exerted an influence on the resistivity. Accordingly, it was essential to specify the resistivity, ρ_∥_ and ρ_⊥_, in relation to the path planning parameters and to limit comparisons to specimens with identical geometries and path planning. Figure 6a illustrates a specimen with a superimposed schematic measurement setup, Figure 6b illustrates the test rig.

Prior to the application of the bonding agent, the thickness of the specimen was measured at both ends and the center with a micrometer screw. The contact mediator was applied to the upper side in the form of 2 mm-wide strips. The two inner measuring contacts were positioned at a distance of 42 mm from one another and were situated 2 mm from the outer feed contacts. The connection between the measuring device and the specimens was established via spring-loaded contacts under conditions that ensured reproducibility. The spring-loaded contacts were arranged in three rows, with the outer rows dedicated to source the current I_FRC_ and the inner one to sense the voltage drop U_SNS_. The contacts of the middle row were not connected. Seven specimens that met the requirements for geometric accuracy were produced from each composite.

#### 2.3.5. Specimen and Measurement Setup for Measuring Layer MEX-Resistivity (z)

**Figure 7:**
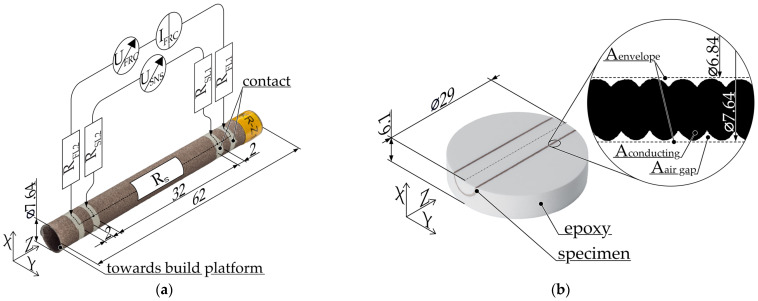
(**a**) Specimen for determining the resistivity in the z-direction; (**b**) specimen to determine the correction factor for the actual electrically conductive specimen cross section. R_S_ = resistance of the specimen, R_FL_ = resistance force lead, R_SL_ = resistance sense lead, I_FRC_ = forced current, U_FRC_ = voltage needed to force current, U_SNS_ = measured voltage drop across specimen, A_envelope_ = enveloping area between the inside and outside of the specimen, A_air gap_ = void areas within the envelope, A_conducting_ = area through which current can flow.

The geometry of the specimen used to determine resistivity along the z-direction differed from that of planar specimens. Manufacturing a vertically standing specimen of that height with a wall thickness of just one strand is not feasible with sufficient reliability. To enhance stability during production, a hollow cylinder was used instead, with a layer height of 200 µm and a nominal wall thickness 400 µm. Daniel et al. employed a similar approach in the production of specimens for determining electrical properties [82]. The outer circumference of the specimen was 24 mm, which was consistent with the width of the planar specimens. This yielded an outer diameter of 7.64 mm for the specimen. The manufacturing was not conducted in the so-called vase mode, which involves the production as a continuous spiral. Rather, the specimen was manufactured in discrete layers, which allowed for a more accurate reproduction of the resulting product’s properties. As the lower layers were not current-carrying during the measurement, minor mechanical defects, such as those resulting from removal from the build platform, could be excluded as sources of error. The number of interlayer contacts was contingent upon the distance between the two measuring contacts and the layer height. Given that the layer height exerted a direct influence on the number of interlayer contacts, it was mandatory to take this into account when interpreting the measurement results. Figure 7a illustrates the specimen with a superimposed measuring circuit, while Figure 7b depicts a specimen for determination of the actual current conducting volume.

Given that the cylindrical specimen has a wall thickness of only one track, it was necessary to consider the discrepancy from the ideal square strand geometry. This is clearly visible in Figure 7b, which illustrates the distortion of a specimen cross section from an ideal cylinder due to convex shapes. Consequently, a correction factor λ was calculated for each composite in order to adjust for the electrically conductive volume.

(2) $$\lambda =\frac{A_{envelope}-A_{gap}}{A_{envelope}}=\frac{A_{conducting}}{A_{envelope}}$$

The correction factor was calculated as the quotient of the actual electrically conductive area A_conducting_ and the desired envelope area A_envelope_. The enveloping area A_envelope_ was calculated for all composites using the nominal outer diameter and the track width. No separate measurement was conducted. The electrically conductive area A_conducting_ was determined through the use of a digital microscope (VHX-7000 with VH-Z20R optic, Keyence, Neu-Isenburg, Germany) with software-based image analysis, thereby eliminating the potential for human bias. As the conductive cross section was determined through destructive analysis, a further z-specimen was produced for each composite and embedded in epoxy (EpoFix, Struers GmbH, Willich, Germany) for the purposes of further analysis. To enhance contrast, a white dye was mixed into the epoxy resin. The specimens were subjected to a vacuum impregnation process for a duration of 30 min at a pressure of 100 mbar absolute. Subsequently, the specimens were cured for a minimum of 48 h, after which they were machined down to the center and sanded with 2500-grit sandpaper (MetaServ 250 GRINDER-POLISHER, Buehler ITW Test & Measurement GmbH European Headquarters, Leinfelden-Echterdingen, Germany). The determined correction factor was subsequently applied to all subsequent specimens of the same composite. The modified Formula (3) was used to calculate the resistivity for the cylindrical specimen, accounting for the volume correction factor λ:

(3) $$\varrho_{P}=R_{P}\cdot \frac{A}{L}\cdot \lambda$$

The electrical contact mediator was applied in the form of 2 mm-wide strips circumferentially. The measurement was conducted using the identical test rig (see Figure 6b) employed for the measurement of the resistance of the planar specimens. Prior to the measurement, a precisely fitting electrically insulating plastic pin, made from polyvinyl chloride (PVC), was inserted into the specimen. This was done in order to minimize specimen deformation due to the force of the spring-loaded contacts. The resistance measurement was performed with a sample size of seven.

## 3. Results

In this section, the identified fillers are compared with the manufacturer’s specifications, and the results of the resistance measurements are presented.

### 3.1. Filler Identification Using Scanning Electron Microscope Micrographs

**Figure 8:**
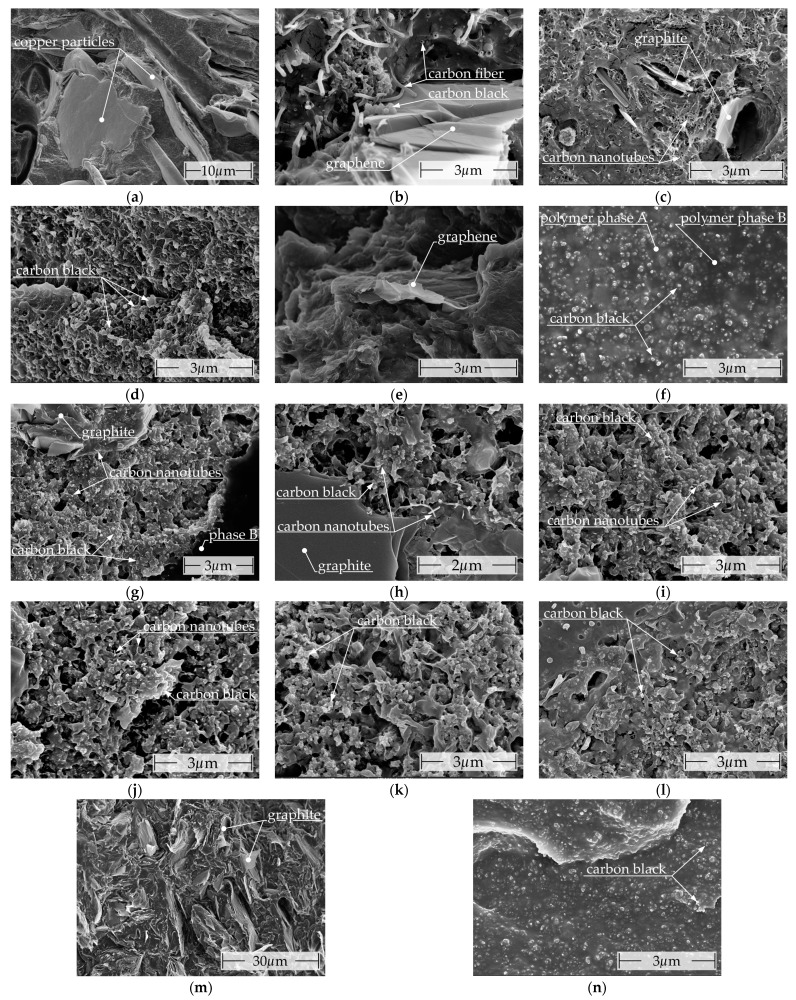
Scanning electron microscope images of the commercially available filaments with marked conductive fillers: (**a**) Multi3d Electrifi; (**b**) BlackMagic Conductive; (**c**) Functionalize F-Electric™ (**d**) Amolen conductive PLA; (**e**) Koltron G1; (**f**) Conductive Filaflex; (**g**) Ampere PLA; (**h**) Alfaohm; (**i**) Fabbrix CNT; (**j**) Nylforce conductive; (**k**) Protopasta conductive PLA; (**l**) 3dkonductive electroconductive; (**m**) FILI conductor; (**n**) Eel 3D-Printer Filament.

The SEM images of the various commercial filaments are presented in Figure 8, arranged in the sequence outlined in Table 1.

**Figure A1:**
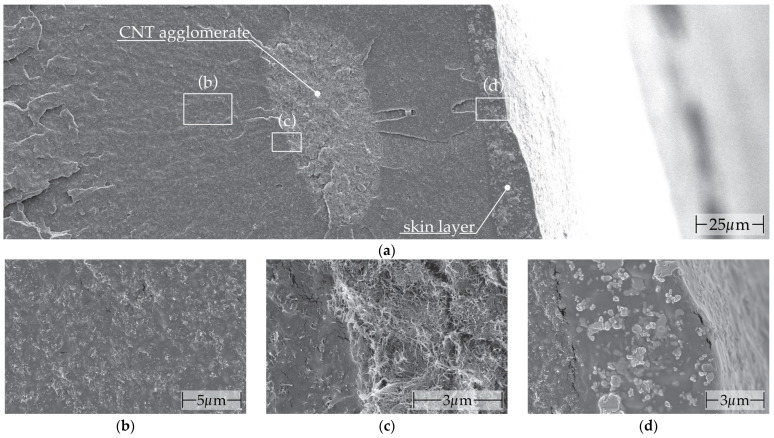
SEM micrographs of a cryofracture surface of a filament from the composite Functionalize F-Electric™ with areas of incompletely solubilized CNTs in the polymer matrix: (**a**) overview; (**b**) polymer matrix with solubilized CNTs; (**c**) boundary area between the area with solubilized and unsolubilized CNTs; (**d**) filament edge.

**Figure A2:**
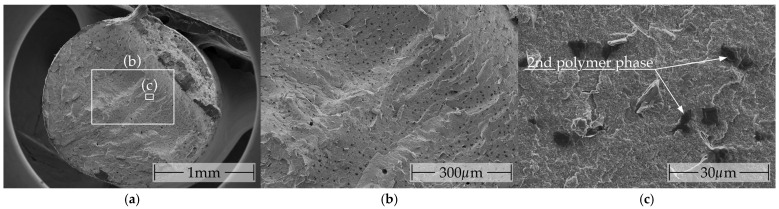
SEM micrographs of the fracture surface of the AmperePLA filament illustrates the homogeneous distribution of the second polymer phase across the entire cross section, evident on different scales (**a**–**c**).

**Figure A3:**
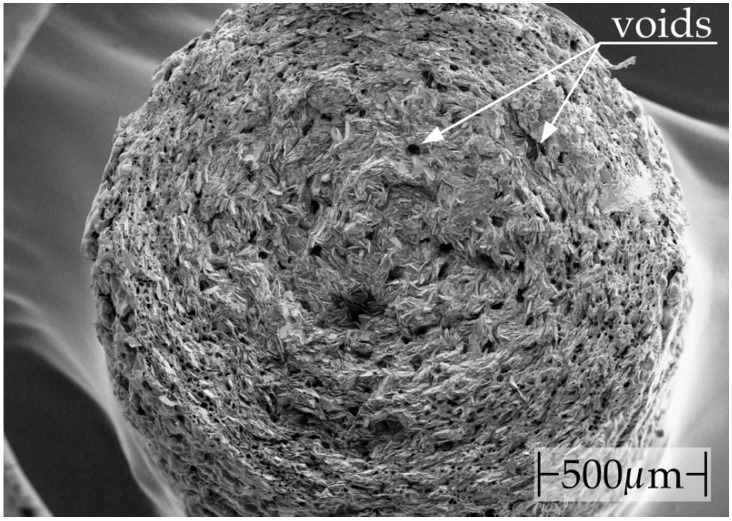
SEM micrographs of fracture surface of Electrifi with a notable degree of porosity.

**Figure A4:**
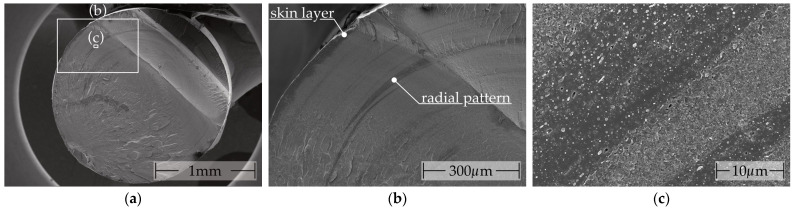
SEM micrographs of the fracture surface of the 3dkonductive electroconductive filament illustrate the inhomogeneous compound of filler within a polymer matrix, which is evident at different scales (**a**–**c**).

**Figure A5:**
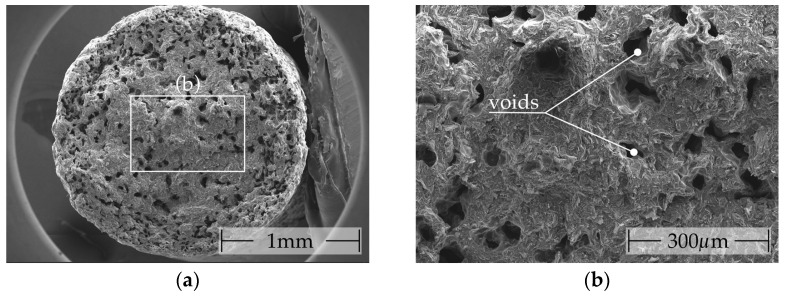
SEM micrographs of fracture surface of FILI conductor with a notable degree of porosity at different scales (**a**,**b**).

The plate-shaped copper particles depicted in Figure 8a are the only conductive fillers utilized in Multi3d Electrifi conductive and are notably larger than the carbon fillers. The filament exhibits a high degree of porosity (see Figure A3). Figure 8b illustrates that BlackMagic conductive comprises carbon black, in addition to graphene and carbon fiber. The carbon fibers with a hollow center exhibit a high degree of similarity to CNTs. Figure 8 provides a visual representation of the synergies that occur during the formation of the conductive network. This is demonstrated through the direct contact of different conductive fillers with varying aspect ratios (length/diameter). Carbon fibers and graphene facilitate direct connections over extended distances, while carbon black connects points in close proximity [83,84,85,86]. As observed in Figure 8c, the conductive network of Functionalize F-Electric™ is predominantly constituted only by CNTs. Individual graphite particles are visible in certain locations. In several locations within the fracture surface, areas with undissolved CNTs (see Figure A1) were observed. In addition, the filament has a different composition on the outside, where no CNTs are visible. The composite Amolen conductive PLA (Figure 8d) contains only CB as a conductive filler. Figure 8e shows the fracture surface of Koltron G1, in which a single graphene particle is visible. In contrast to the smooth fracture surface observed for unfilled PVDF [87], the serrated fracture surface of the composite indicates that the majority of the graphene is well dissolved. No other fillers are evident. Figure 8f illustrates two distinct polymer phases of the flexible TPU composite Conductive Filaflex. Phase A, the lighter phase, readily dissolves the conductive filler CB. In contrast, phase B, the darker phase, contains minimal to no CB. Ampere PLA (Figure 8g) features the inclusion of CB and graphite as conductive fillers, in addition to the specified CNT. Moreover, another polymer phase (B) is identifiable, which exhibits a homogeneous distribution as inclusions within the primary phase (see Figure A2). This phase does not contain any fillers. In addition to carbon nanotubes and carbon black, Alfaohm (Figure 8h) also contains graphite as an electrically conductive filler. Contreras-Naranjo et al. conducted a more detailed investigation of the composition and determined a proportion of approximately 3%wt. MWCNT with a 1:10 CNT/CB ratio [88]. The SEM micrographs of Fabbrix CNT (Figure 8i) and Nylforce conductive (Figure 8j) exhibit notable similarities. In addition to CNTs, both composites also contain carbon black and amorphous graphite. Protopasta conductive PLA, as illustrated in Figure 8k, contains exclusively carbon black as filler. The conductive network in 3dkonductive electroconductive (Figure 8l) is formed exclusively of carbon black. As illustrated in Figure A4, radial areas with a markedly low CB content suggest insufficient compounding. These isolating areas are also widespread on the outside of the filament. Of the composites examined, FILI conductor (Figure 8m) is the only one that contains graphite in a high concentration as a conductive filler. This makes the material highly brittle, despite its TPU/PLA matrix, and it lacks the flexibility observed in other TPU-based composites, such as Eel 3D-Printer Filament or Conductive Filaflex. No additional conductive fillers could be identified. The filament exhibits a high degree of porosity (see Figure A5). The composite Eel 3D-Printer Filament (see Figure 8n) contains only carbon black. It is evident that a considerable number of manufacturers of CNT-containing composites incorporate additional conductive fillers, such as carbon black (Ampere PLA, Alfaohm, Fabbrix CNT, Nylforce conductive) [83,84,85,86] or graphite (Alfaohm, Functionalize F-Electric™, Ampere PLA), with the objective of enhancing conductivity.

It should be noted that surface layers are not unique to Functionalize F-Electric™ and 3dkonductive filaments but are likely to occur in other composites as well. In addition to being identified by their distinct texture and composition, there may also be a gradual depletion of conductive fillers in these areas. However, this depletion is not visible in the SEM micrographs due to the high surface conductivity created by the platinum sputtering process used for imaging. Table 3 presents a summary of the findings derived from the SEM micrographs.

### 3.2. Electrical Resistivity

Table 4 lists the results of the measurements conducted on the filament feedstock, fibres and the manufactured MEX specimen.

As previously detailed, a direct comparison between the resistivities of the distinct MEX specimens (ρ_∥_, ρ_⊥_, ρ_z_) is not possible. This also applies to values determined using alternative specimen geometries, as described in the literature. However, available literature values are provided as a reference.

The conductivities declared by the manufacturers were replicated in a comparable order of magnitude or better for the majority of composites. Exceptions to this are Conductive Filaflex, Amolen conductive PLA, 3D Conductive, and FILI Conductor TPU.

The conductivity of the majority of composites remains unaltered or exhibits a decline of up to 221% (Conductive Filaflex) as a consequence of extrusion into fiber. In contrast, extrusion has a notable impact on the resistivity of FILI Conductor, decreasing the resistivity from 220.2 Ωcm for filament to 35.78 Ωcm for fibers and for 3dkonductive electroconductive from 48.18 Ωcm to 15.96 Ωcm, respectively. This may be attributed to a reduction in inhomogeneities in 3dkonductive (see SEM micrograph of 3dk filament, Figure A4), and a reduction in porosities in FILI Conductor (see SEM micrograph of FILI Conductor filament, Figure A5). Furthermore, the standard deviation of the fiber specimens is lowest (<3%) for composites based on CNT or graphene.

The conductivity of all composites is negatively affected by the additive processing of filament to MEX structures. Similar results were also observed for a composite of acrylonitrile butadiene styrene (ABS) with graphene (ABS/G) [79], for PLA/CB [53] and PLA/CNT [89].

The resistivities of the CNT/CB/x composites Alfaohm, Ampere PLA, Fabbrix CNT, and Nylforce conductive exhibit a high degree of similarity within the individual specimen geometries. In contrast, the graphene-containing composites Koltron G1 (PVDF/G) and Blackmagic Conductive (PLA/G/CF/CB) do not exhibit any notable similarities within a specimen geometry. The presence of additional fillers in Blackmagic, in addition to graphene, and the use of different matrix polymers may be contributing factors. The purely CB-containing composites (Protopasta conductive PLA, Conductive Filaflex, Amolen conductive PLA, and 3D conductive conductor) display a notable range of resistivity values for the individual specimens. Furthermore, the standard deviation is in average greater than that observed for the remaining composites.

A quantitative comparison of the results of individual specimen geometries is not possible due to the influence of uncompensated factors. Nevertheless, some tendencies resulting from the initial factors can be derived. It was observed that composites containing CNT/CB exhibited a relatively constant resistivity regardless of the direction of current flow with respect to the strands. It was observed that composites filled exclusively with CB demonstrated higher conductivity along the z-direction compared to the planar (∥, ⊥) ones. An exception to this trend was identified with 3dkonductive electroconductive.

Of the investigated composites, the copper-filled composite Multi3d Electrifi is the most conductive, displaying a resistance of 0.021 ± 0.003 Ωcm, which can be attributed to the high conductivity of the copper filler. The measured value is consistent with the results of Flowers et al. and Watschke et al., who reported ϱ_∥,lit_ = 0.014 Ωcm [25] and 0.0286 Ωcm [21], respectively. Furthermore, Watschke et al. determined ρ_⊥,lit_ = 0.0678 Ωcm. It should be noted that Multi3d Electrifi also exhibits a substantial degree of variability across all specimens.

The Blackmagic Conductive sample displays the most significant relative standard deviation among the graphene and CNT-containing composites with ϱ_⊥_ = 4.707 ± 0.824 Ωcm (±17.51%) and ϱ_z_ = 9.69 ± 1.311 Ωcm (±13.53%). Furthermore, the resistivity in the z-direction is relatively high compared to the other composites with high aspect ratio fillers. The observed values are consistent with previously reported literature data for ϱ_∥,lit_ = 0.78 Ωcm [25] and ϱ_z,lit_ =12 to 16 Ωcm [51].

Protopasta conductive PLA exhibits the highest conductivity of the purely CB-containing composites, with a resistivity of 11.69 ± 0.28 Ωcm. The standard deviation of resistivity for all specimen geometries is less than ±2.5%. The resistivity of the filament samples is comparable to the values reported by Stankevich et al., with a value of ρ_fila,lit_ = 4.971 ± 0.046 [53]. In previous studies, the resistivity of MEX structures has been measured to be 6 Ωcm (Lazarus et al.), 12 Ωcm (Flowers et al.), and 10.65 Ωcm (Watschke et al.) [21,25,57]. Gao et al. determined the resistivity in the xy-plane with an unclear infill orientation to be 10 to 13 Ωcm, while the resistivity in the z-direction was observed to be between ϱ_z_ = 20 and 45 Ωcm for layer thicknesses ranging from 200 to 600 μm [51].

All MEX specimens made of 3dkonductive electroconductive that were examined exhibit a high standard deviation (∥ = ±24.17%, ⊥ = ±52.46%, z = ±26.60%).

An examination of the composites revealed that FILI Conductor exhibited the lowest conductivity in the majority of categories, accompanied by a notable elevation in the standard deviation across all measurements. Despite being a composite with a flexible TPU as the matrix polymer, the resulting composite exhibits brittle mechanical properties, thereby rendering it very challenging to process.

The resistivity of the Koltron G1 filament samples is comparable to the values reported by Stankevich et al. (ϱ_fila,lit_ = 3.36 ± 0.08 Ωcm) [53].

## 4. Discussion

In order to ensure comparability of the resistivity of the MEX specimens, the MEX measurement results are normalized to the resistivity of the fiber of the respective composite. For further details, please refer to Table A2. The following trends emerge from the results with regard to the influence of the path planning (independent of fillers) on the anisotropy of the resistivity of the MEX specimens:

1. Fibers are less conductive than filament.
2. The MEX specimens exhibit lower conductivity than filament and fiber.
3. The conductivity of MEX structures is highest along the strands.
4. The conductivity of MEX structures in the z-direction is superior to that within the xy-plane perpendicular to the strand direction.

These observations can be explained by a depletion of conductive fillers in the skin layer, which results in a locally reduced electrical conductivity. A depletion of the surface layer is a well-documented phenomenon in the extrusion of filled composites [53,61,79,90,91]. A comparison of the outer areas of the filaments with the core of SEM micrographs of Functionalize F-Electric™ (see Figure A1) and 3dkonductive electroconductive (see Figure A4) filaments reveals notable differences between them. These differences can be attributed to the manufacturing process of the filament. Similar findings have been reported by Stankevich et al. and Wolterink et al. [53,61]. Abdalla et al. propose that the depletion of the skin layer can be attributed to density differences between the fillers and the matrix polymer, which results in a concentration of the fillers in the core [91]. Another potential influencing factor is the expansion of the polymer after leaving the nozzle (so-called die swell), which forms an insulating layer of polymer [79,90].

**Figure 9:**
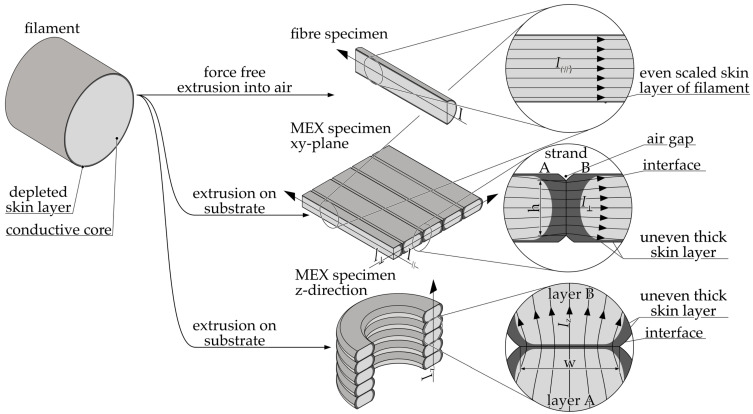
A schematic representation of the formation of the anisotropic skin layer based on the simplified assumption of an abrupt transition from conductive to insulating properties. I = current flow in fiber; I_∥_ = current flow parallel to the strands; I_⊥_ = current flow orthogonal to the strands; I_z_ = current flow through layers; h = contact height between strands; w = contact width between layers.

Figure 9 provides a schematic illustration of the propagation of the influence of the skin layer from the filament to the fiber and MEX structure. This ultimately results in the formation of a conductive network in MEX structures with anisotropic electrical properties due to the different skin layer thicknesses.

The extrusion of filament through a MEX nozzle does not result in further homogenization of the conductive fillers in the melt, due to the laminar flow of the polymer melt. Re-extrusion during MEX processing promotes the formation of a skin layer, as the effects that also cause a depletion of the conductive fillers in the skin layers during filament production have the same effect again. Given that the cross-sectional geometry of the fiber is similar to that of the filament, the skin layer is transferred to the fiber in a scaled form but remains geometrically unchanged. The impact of the reduced conductivity of the skin layer is compensated for during filament and fiber measurements through the utilization of a four-wire measurement technique, the employment of considerable distances between the electrical contacts, and the current flow within the same strand. A slight decline in conductivity is observed in the fibers in comparison to the filament, which can be attributed to the destruction of conductive networks, such as CB agglomerates, and a concentration of fillers within the core through re-extrusion. One exception is the FILI conductor, which has demonstrated an increase in conductivity that may be attributed to a reduction in porosity (see SEM micrograph of FILI Conductor filament, Figure A5).

**Figure 10:**
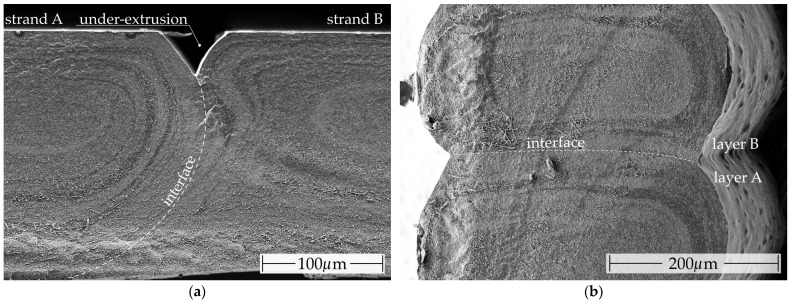
Scanning electron microscope (SEM) micrographs of a cryo-fractured MEX specimen comprising 3D conductive electroconductive, which exhibit visible differences in the thicknesses of the various phases observed along the strand cross section (left/right, top/bottom): (**a**) fracture surface through the xy-plane of a planar MEX specimen; (**b**) fracture surface along the z-direction of a MEX specimen.

The reduced conductivity of the skin layer is particularly responsible for the anisotropic electrical properties of MEX structures. In contrast to the fabrication of fibers, the melt flow in the additive manufacturing of MEX structures is constrained by the underlying and laterally deposited strands. The cross section of the strands is approximately rectangular in geometry. The aspect ratio of a strand is defined as the ratio of layer height to track width and is typically 50% [92,93]. This yields edge layers that are broader on the lateral surfaces than on the top and bottom of the strands. Figure 10a,b illustrates the deformation observed on the left and right strand edges and on the upper and lower sides, respectively.

The SEM micrographs in Figure 10b illustrate especially that the discrepancy in material composition between the strand on the right-hand side and the bottom or top side is significantly more pronounced. It is evident from both figures that the radial pattern observed in the filament cross section shown in Figure A4 is transferred to the strands as a consequence of laminar flow, without further homogenization by the extrusion.

The resistivity is subject to influence from the direction of current flow in relation to the orientation of the strands. A current flow parallel to the strands (I_∥_) is minimally impeded by the less-conductive skin layers. Moreover, research has shown that the conductive fillers can be oriented in alignment with the extrusion direction [40,42,89,94,95,96,97]. The rapid cooling of the strands subsequent to deposition results in the formation of a frozen state of the melt and the fillers dissolved therein [90]. Composites comprising fillers with a high aspect ratio are particularly susceptible to this phenomenon, resulting in enhanced conductivity along the strands. To illustrate, the composite Functionalize F-Electric™, which is predominantly filled with high aspect ratio CNTs, exhibits a resistivity that is 348.33% higher in the direction perpendicular to the strands in the xy-plane than in the direction parallel to the strands. In contrast to high aspect ratio fillers, rounded fillers such as CB demonstrate minimal alignment, as particles shape exhibits no discernible preference along a flow profile. As an example, the Eel 3D printing filament shows almost no difference in normalized resistivity between specimens with parallel and perpendicular infill orientations. Similarly, the difference for Protopasta Conductive PLA (140.03%) and Conductive Filaflex (152.67%) is minimal when compared to Functionalize F-Electric™. Notably, composites containing both carbon black and CNTs, such as Ampere PLA, Fabbrix CNT, and Nylforce Conductive, display a significantly smaller difference between normalized resistivity for parallel and perpendicular measurements compared to the CNT-only composite, Functionalize F-Electric™. As a result of these effects, the resistivity is lowest in the direction parallel to the strands for all studied composites.

Conversely, when the flow direction is perpendicular to the strand (I_⊥_), the current will invariably flow through the skin layers. This is applicable to samples exhibiting a filling pattern orientation that is perpendicular to the current flow within the xy-plane and along the z-direction. The results demonstrate that the resistivity normalized to the fiber in the z-direction is lower for the majority of composites than in the perpendicular xy-plane. The contact width (w) between the strands in the z-direction is greater than the contact height (h) of the strands within a layer. The larger contact area between the strands in the z-direction facilitates the welding of the strands, as the larger contact area enhances the heat transfer after the melt application, allowing the surface of the neighboring strands to be melted more rapidly before the temperature falls below the melting temperature. The melting of the neighboring strands may result in enhanced cross-linking of the conductive networks in the adjacent strands. In the xy-plane, a slight under-extrusion occurs at the contact points between the strands, which is an intentional aspect of the manufacturing process to ensure accurate thickness measurement [24]. Similarly, a bulbous profile forms along the z-direction on the strand sides due to the production process. This results in a constriction of the electrically conductive cross section at the contact points [11]. As a consequence of the geometric deviation, the actual cross-sectional area is smaller than the ideal area used in Equation (1) to calculate the resistivity. This results in a higher resistivity than would otherwise be the case. For measurements taken in the z-direction, the resulting error is considerable; however, the correction factor from Equation (2) is utilized to compensate this effect. Given that the geometric deviation within the samples used to determine the resistivity in the xy-plane is minimal, no compensation was deemed necessary. Consequently, the resistivity in the xy-plane is slightly higher than the actual resistivity. As the contact area in the z-direction is larger, the welding is better and the skin layer is less pronounced, which leads to a lower resistivity in this direction.

In the z-direction, nearly half (6/14) of all composites exhibit a standard deviation of less than ±2.5% of the mean resistivity (∥: 2/14, ⊥: 2/14). One potential explanation for the reduced standard deviation is the method of polymer deposition. The initial layer serves to compensate for minor unevenness, thereby ensuring constant layer thicknesses for all subsequent layers. The findings indicate that incorporating conductive fillers with a high aspect ratio significantly enhances the electrical contact between layers, leading to a reduction in resistance. However, there are exceptions to this, including Protopasta conductive PLA, which, as a purely CB-containing composite, also exhibits a standard deviation of less than 2.5% in the z-direction. In contrast, both Blackmagic Conductive and Functionalize F-Electric™, despite containing graphene or CNT, display a standard deviation along the z-direction exceeding 2.5%.

In addition to the placement of the strands, the composition of the composite has an influence on the resistivity. In the case of conventional processing of composites comprising CB (e.g., injection molding), it is reported that a filler concentration closer to the percolation threshold leads to a higher affection of the resistivity by the processing influences [98]. At the percolation threshold, a minor variation in filler concentration results in a significant change in resistivity. It seems reasonable to conclude that the composites exhibiting the highest standard deviations are those with the lowest CB concentrations. Among the CB-containing composites, Protopasta conductive PLA exhibits the most notable conductivity with the lowest standard deviation. In addition, poor compounding, as exemplified by 3dkonductive electroconductive (see Figure A4), can also result in a high standard deviation due to a significant variation in the formation of the conductive network within the phases of good and bad conductivity.

In contrast to composites comprising carbon allotropes as fillers, the resistivity of metal-filled composites is susceptible to temperature-induced oxidation effects during extrusion. This could have a considerable impact on the high standard deviation observed in Electrifi [21,65].

## 5. Conclusions

This study presents a quantitative data basis for a comparative analysis of the resistivity of a total of 14 commercially available filaments for material extrusion. For the initial step, the fillers were identified through the use of scanning electron microscopy imaging. Subsequently, the resistivity of the filament and fibers extruded from it, as well as MEX structures, was determined, with consideration of the anisotropic electrical properties. The key findings include the following:

1. Some filaments have a distinctly different composition and texture on the outside than on the inside.
2. A significant number of composite materials utilizing graphene or CNTs as a conducting filler also employ CB, although this is not specified by the manufacturer.
3. The conductivity of the composites is reduced by manufacturing MEX structures from filament.
4. Composites with metallic conductive filler exhibit the highest conductivity, followed by those containing CNTs and graphene.
5. Composites with high aspect ratio fillers exhibit a low standard deviation across all specimen types.
6. The resistivity of MEX-structures shows the lowest standard deviation along the z-axis.

The data were examined for patterns and a hypothesis was formulated, based on the findings, which suggests that the differences can be explained by the formation of a skin layer with a lower conductivity.

In selecting and designing products using these composites, it is essential to consider additional electrical properties, such as impedance, piezoresistive behavior, and temperature-dependent resistivity. These properties, in addition to resistivity measured at room temperature, are crucial for material selection and product design, depending on the intended use. Further studies are required to cover these aspects in a comparable manner. In order to integrate the multi-material system into a product, further knowledge of the mechanical bond and the electrical contact resistance between several electrical functional structures is necessary. Additionally, the interface with conventional electrical conductors must be investigated from both an electrical and mechanical perspective.

## Figures and Tables

**Table 1:** Overview of commercially available electrically conductive filaments as per manufacturer specifications. The composites are sorted in ascending order of their resistivity.

Composite Name	Matrix Polymer	Fillers	Concentration [% wt]	Resistivity [Ω·cm]
Multi3d Electrifi [65]	PCL	CuP	>1	0.006
BlackMagic Conductive [66]	PLA	G CF	30–40 10	0.6
Functionalize F-Electric™ [67]	PLA	CNT	n.a.	0.75
Amolen conductive PLA [68]	PLA	n.a.	n.a.	1.42
Koltron G1 [69]	PVDF	G	<10	2
Conductive Filaflex [70]	TPU	CB	n.a.	3.9
Ampere PLA [71]	PLA	CNT	n.a.	4
Alfaohm [72]	PLA	CNT, CB	n.a.	15 (xy)/20 (z)
Fabbrix CNT [73]	n.a.	CNT	n.a.	n.a.
Nylforce conductive [74]	PLA	n.a.	n.a.	n.a.
Protopasta Conductive PLA [75]	PLA	CB	<21.43	30 (xy)/115 (z)
3dkonductive electroconductive [76]	PLA/TPU	CB	n.a.	24
FILI conductor [77]	TPU	n.a.	n.a.	27.44
Eel 3D-Printer Filament [78]	TPU	CB	<18	1500

Matrix polymers: PCL = polycaprolactone; PLA = polylactic acid; PVDF = polyvinylidene fluoride, TPU = thermoplastic polyurethane. Fillers: CuP = copper particles; CF = carbon fiber, G = graphene; CNT = carbon nanotube (no distinction between single and multiwall CNTs); CB = carbon black.

**Table 2:** Process parameters used for additive fabrication of the MEX specimen.

Composite Name	Extrusion Temperature [° C]	Build Plate Temperature [° C]	Speed for Planar Specimen [mm/s]	Speed for z-Specimen [mm/s]	Electrical Bonding Agent
Multi3d Electrifi [65]	140	RT	20	5	EMS#12640
BlackMagic Conductive [66]	220	50	30	10	EMS#12640
Functionalize F-Electric™ [67]	222	40	30	10	EMS#12640
Amolen conductive PLA [68]	235	RT	35	10	EMS#12640 ^1,2^, 8331D-14G ^3^
Koltron G1 [69]	287	60	15	10	EMS#12640 ^1,2^, 8331D-14G ^3^
Conductive Filaflex [70]	250	55	20	10	EMS#12640
Ampere PLA [71]	235	50	25	10	EMS#12640
Alfaohm [72]	215	60	30	10	EMS#12640
Fabbrix CNT [73]	215	40	62	10	EMS#12640
Nylforce conductive [74]	215	40	62	10	EMS#12640
Protopasta Conductive PLA [75]	210	60	35	10	EMS#12640
3dkonductive electroconductive [76]	215	65	40	10	EMS#12640
FILI conductor [77]	260	RT	40	10	EMS#12640
Eel 3D-Printer Filament [78]	230	32	15	10	None ^2^, EMS#12640 ^1,3^

^1^ = Bonding agent used with fiber specimen; ^2^ = Bonding agent used with filament specimen; ^3^ = Bonding agent used with MEX specimen; RT = room temperature 23 ± 1 ° C.

**Table 3:** Overview of the fillers included in the composite, as stated by the manufacturer, and the electrical conductive fillers actually included.

Composite Name	Fillers according to Manufacturer	Additionally Discovered Fillers
Multi3d Electrifi [65]	CuP	none
BlackMagic Conductive [66]	graphene, CF	CB
Functionalize F-Electric™ [67]	CNT	(Gr)
Amolen conductive PLA [68]	n.a.	CB
Koltron G1 [69]	G	none
Conductive Filaflex [70]	CB	none
Ampere PLA [71]	CNT	CB, Gr
ALFAOHM [72]	CNT, CB	Gr
Fabbrix CNT [73]	CNT	CB, Gr
Nylforce conductive [74]	CNT	CB, Gr
Protopasta Conductive PLA [75]	CB	none
3dkonductive electroconductive [76]	CB	none
FILI conductor [77]	n.a.	Gr
Eel 3D-Printer Filament [78]	CB	none

Fillers: CuP = copper particles; CF = carbon fiber, G = graphene; CNT = carbon nanotube (no distinction between single and multiwall CNT); CB = carbon black; Gr = graphite.

**Table 4:** Resistivity at room temperature (RT = 23 ± 1 °C) for filaments, fibers, and MEX structures with a strand width of 400 µm and a layer height of 200 µm. Please refer to Table A2 for the complete version of this table, which includes the percentage standard deviation.

Composite Name	Resistivity according to Manufacturer [Ωcm]	Resistivity of Filament [Ωcm]	Resistivity of Fiber [Ωcm]	Resistivity ϱ_∥_ [Ωcm]	Resistivity ϱ_⊥_ [Ωcm]	Resistivity ϱ_z_ [Ωcm]
Multi3d Electrifi	0.006 [65]	0.013 ± 7 × 10^−4^	0.013 ± 8 × 10^−5^	0.021 ± 0.003	0.048 ± 0.003	0.061 ± 0.008
BlackMagic Conductive	0.6 [66]	0.795 ± 0.052	0.832 ± 0.023	0.981 ± 0.032	4.707 ± 0.824	9.690 ± 1.311
Functionalize F-Electric™	0.75 [67]	0.532 ± 0.015	0.761 ± 0.015	1.109 ± 0.112	3.863 ± 0.114	2.133 ± 0.088
Amolen conductive PLA	1.42 [68]	22.33 ± 0.15	25.59 ± 0.777	111.3 ± 9.880	320.3 ± 39.75	87.77 ± 13.56
Koltron G1	2 [69]	3.39 ± 0.01	3.238 ± 0.029	7.287 ± 0.299	10.09 ± 0.145	5.741 ± 0.104
Conductive Filaflex	3.9 [70]	9.10 ± 0.20	20.18 ± 0.949	34.88 ± 6.637	53.25 ± 11.33	22.39 ± 2.593
Ampere PLA	4 [71]	1.80 ± 0.03	2.015 ± 0.050	3.091 ± 0.263	4.606 ± 0.663	3.707 ± 0.064
Alfaohm	15(xy)/20(z) [72]	1.70 ± 0.01	3.090 ± 0.035	3.614 ± 0.066	7.052 ± 0.248	6.631 ± 0.125
Fabbrix CNT	n.a.	1.80 ± 0.01	3.037 ± 0.049	4.543 ± 0.220	6.006 ± 0.494	5.720 ± 0.014
Nylforce conductive	n.a.	3.752 ± 0.033	4.179 ± 0.028	8.348 ± 0.701	11.07 ± 0.501	8.491 ± 0.164
Protopasta conductive PLA	30(xy)/115(z) [75]	6.53 ± 0.06	6.216 ± 0.014	11.69 ± 0.282	16.37 ± 0.385	8.005 ± 0.199
3dkon. electroconductive	24 [76]	48.18 ± 0.73	15.96 ± 1.277	172.5 ± 41.69	1216 ± 637.9	365.9 ± 97.35
FILI conductor	27.44 [77]	220.2 ± 49.9	35.78 ± 6.056	242.9 ± 51.47	685.9 ± 64.76	162.3 ± 12.58
Eel 3D Printer Filament	1500 [78]	10.63 ± 0.14	19.84 ± 3.966	90.77 ± 27.92	91.96 ± 29.51	27.78 ± 7.859

ϱ_∥_ = resistivity measured with current flow parallel (0°) to the orientation of infill pattern; ϱ_⊥_ = resistivity measured with current flow perpendicular (90°) to the orientation of infill pattern; ϱ_z_ = resistivity measured with current flow through the layers.

## Data Availability

The data presented in the study, along with the figures and SEM micrographs, are openly available at FigShare at https://doi.org/10.6084/m9.figshare.25805269.v2.

## Appendix A

**Table A1:** Summary of the process parameters specified by the manufacturer and the parameters chosen for sample production.

Composite Name	Extrusion Temperature [°C]			Build Plate Temp. [°C]			Speed [mm/s]			
	Min.	Max.	Chosen	Min.	Max.	Chosen	Min.	Max.	Chosen	
									xy	**z**
Multi3d Electrifi [65]	140	160	140 ^2^	n.a.	n.a.	RT	10	30	20	5
BlackMagic Conductive [66]	220	220	220	50	50	50	30	30	30	10
Functionalize F-Electric™ [67]	215	230	222	RT	70	40	n.a.	n.a.	30	10
Amolen conductive PLA [68]	220	250	235	RT	50	35	30	70	50	10
Koltron G1 [69]	260/280 ^1^	285/295 ^1^	287	n.a./60 ^1^	100/60 ^1^	60	20	20	20	10
Conductive Filaflex [70]	245	250	250 ^2^	50	60	55	20	20	20	10
Ampere PLA [71]	210	250	235 ^2^	40	60	50	20	30	25	10
Alfaohm [72]	190	210	215 ^2^	RT	50	60 ^2^	10	50	30	10
Fabbrix CNT [73]	215	215	215	40/30 ^1^	85/50 ^1^	40 ^2^	30/40 ^1^	50/85 ^1^	62 ^2^	10
Nylforce conductive [74]	215	215	215	30	50	40	40	85	62	10
Protopasta Conductive PLA [75]	215/195 ^1^	215/225 ^1^	210	60	60	60	25	45	35	10
3dkonductive electroconduc. [76]	200	230	215	60	70	65	n.a.	90	40	10
FILI conductor [77]	250	260	260 ^2^	n.a.	n.a.	RT	40	40	40	10
Eel 3D-Printer Filament [78]	220	230	230 ^2^	RT	45	32	15	20	15	10

^1^ = Conflicting (datasheet or website) process parameter with filament spool, ^2^ = No use of the mean parameter value (see Section 2.1), RT = room temperature 23 ± 1 °C.

**Table A2:** Overview of the measured values of the filament, fiber, and MEX samples with percentual standard deviation (SD) and normalization of the measured values to the resistivity of the fiber. The composites are sorted in descending order according to the resistivity of the filament. ϱ_fila_ = filament resistivity, ϱ_fiber_ = resistivity fiber, ϱ_∥_ = resistivity of MEX specimen with current flow parallel to strands, ϱ_⊥_ = resistivity of MEX specimen with current flow perpendicular to strands, ϱ_z_ = resistivity of MEX specimen with through the layer, MV = mean value, SD = standard deviation.

Composite Name	Resistivity ϱ_fila_		Resistivity ϱ_fiber_		Resistivity ϱ_∥_		Resistivity ϱ_⊥_		Resistivity ϱ_z_		Normalised to Fiber [%]			
	MV ± SD [Ωcm]	SD [%]	MV ± SD [Ωcm]	SD [%]	MV ± SD [Ωcm]	SD [%]	MV ± SD [Ωcm]	SD [%]	MV ± SD [Ωcm]	SD [%]	$\frac{\varrho_{fila}}{\varrho_{fiber}}$	$\frac{\varrho_{∥}}{\varrho_{fiber}}$	$\frac{\varrho_{⊥}}{\varrho_{fiber}}$	$\frac{\varrho_{z}}{\varrho_{fiber}}$
Multi3d Electrifi	0.013 ± 7 × 10^−4^	5.38	0.013 ± 8 × 10^−5^	0.61	0.021 ± 0.003	14.2	0.048 ± 0.003	6.25	0.061 ± 0.008	13.11	99.24	160.3	366.4	465.6
Functionalize F-Electric™	0.532 ± 0.015	2.81	0.761 ± 0.015	2.01	1.109 ± 0.112	10.0	3.863 ± 0.114	2.95	2.133 ± 0.088	4.12	69.84	145.5	507.0	280.0
BlackMagic Conductive	0.795 ± 0.052	6.54	0.832 ± 0.023	2.80	0.981 ± 0.032	3.26	4.707 ± 0.824	17.5	9.690 ± 1.311	13.52	95.51	117.8	565.4	1164
Alfaohm	1.70 ± 0.01	0.58	3.090 ± 0.035	1.14	3.614 ± 0.066	1.82	7.052 ± 0.248	3.51	6.631 ± 0.125	1.88	55.00	116.9	228.1	214.5
Ampere PLA	1.80 ± 0.03	1.66	2.015 ± 0.050	2.48	3.091 ± 0.263	8.50	4.606 ± 0.663	14.3	3.707 ± 0.064	1.72	89.30	153.3	228.5	183.9
Fabbrix CNT	1.80 ± 0.01	0.55	3.037 ± 0.049	1.62	4.543 ± 0.220	4.84	6.006 ± 0.494	8.22	5.720 ± 0.104	1.81	59.27	149.5	197.7	188.3
Koltron G1	3.39 ± 0.01	0.29	3.238 ± 0.029	0.90	7.287 ± 0.299	4.10	10.09 ± 0.145	1.43	5.741 ± 0.041	0.71	104.6	225.0	311.5	177.2
Nylforce conductive	3.752 ± 0.033	0.87	4.179 ± 0.027	0.66	8.348 ± 0.701	8.39	11.07 ± 0.501	4.52	8.491 ± 0.164	1.93	89.76	199.7	264.8	203.1
Protopasta conductive PLA	6.53 ± 0.06	0.91	6.216 ± 0.014	0.22	11.69 ± 0.282	2.41	16.37 ± 0.385	2.35	8.005 ± 0.199	2.48	105.0	188.0	263.3	128.7
Conductive Filaflex	9.10 ± 0.20	2.19	20.18 ± 0.949	4.70	34.88 ± 6.637	19.0	53.25 ± 11.33	21.2	22.39 ± 2.593	11.57	45.08	172.7	263.8	110.9
Eel 3D Printing Filament	10.63 ± 0.14	1.31	19.84 ± 3.966	19.9	90.77 ± 27.92	30.7	91.96 ± 29.51	32.0	27.77 ± 7.859	28.29	53.57	457.4	463.4	140.0
Amolen conductive PLA	22.33 ± 0.15	0.67	25.59 ± 0.777	3.03	111.3 ± 9.880	8.87	320.3 ± 39.75	12.4	87.77 ± 13.56	15.45	87.24	434.8	1251	342.8
3dkonductive electroconductive	48.18 ± 0.73	1.51	15.96 ± 1.277	7.99	172.5 ± 41.69	24.1	1216 ± 637.95	52.4	365.9 ± 97.35	26.59	301.7	1080	7616	2292
FILI conductor TPU	220.2 ± 49.9	22.6	35.78 ± 6.056	16.9	242.9 ± 51.47	21.1	685.9 ± 64.76	9.44	162.3 ± 12.57	7.745	615.4	678.8	1916	453.7
